# Effects of milk fat globule membrane ingestion with exercise on physical strength in healthy young adults: a randomized double-blind, placebo-controlled trial

**DOI:** 10.1080/15502783.2025.2535372

**Published:** 2025-07-20

**Authors:** Kyosuke Nakayama, Kyoko Ito, Yoshihiko Minegishi, Noriyasu Ota, Shukuko Ebihara, Chiaki Sanbongi

**Affiliations:** a R & D Division, Meiji Co., Ltd., Hachiouji, Tokyo, Japan; b Kao Corporation, Biological Science Research, Haga-gun, Tochigi, Japan; c Chiyoda Paramedical Care Clinic, Chuo-ku, Tokyo, Japan

**Keywords:** Word, muscle strength, agility, muscle power, sphingomyelin

## Abstract

**Background:**

The milk fat globule membrane (MFGM) is a structural membrane that covers the globules of triglycerides dispersed in an emulsion of milk. In previous human studies, MFGM ingestion combined with light aerobic exercise training improved agility in elderly individuals. The purpose of this study was to examine whether 4 weeks of daily ingestion of MFGM combined with power training improves instantaneous physical strength – muscle strength, agility, and muscle power compared with placebo (whey protein isolate) in healthy young adults.

**Method:**

The study was designed as a randomized, double-blind, and placebo-controlled trial. Ninety-eight healthy young adults aged 20–49 completed the study intervention, and they received either an MFGM powder containing 1.7 g of fat and 160 mg of sphingomyelin or an isocaloric placebo powder daily throughout 4 weeks (3 times/week) of power training. Physical strength tests and body composition measurements were conducted before and after the four-week intervention. An analysis of covariance (ANCOVA) model with baseline values as a covariate was carried out to test differences in post intervention values between groups.

**Results:**

Ingestion of MFGM significantly improved peak torque (*p* = 0.003) and average power (*p* = 0.019) of isokinetic knee extensors, leg press 1 repetition maximum (RM) (*p* = 0.004) and sit-ups reps (*p* = 0.030), but not indicators of agility, compared with placebo after the 4-week intervention. There were no significant changes in lean body mass during the intervention period in either group and no significant differences between groups.

**Conclusions:**

We conclude that daily MFGM supplementation combined with power training are effective to improve instantaneous physical strength, such as muscle strength and power, compared with placebo in healthy young adults.

## 1. Introduction

Physical strength declines with age. In accordance with two large-scale cohort studies [1,2] and a meta-analysis [3], muscle strength has been reported to peak in the 20s and 30s, remain stable or decline slowly into the 40s, and decline clearly after 50 years of age. Especially, physical strength during quick movements such as chair standing [4], jumping [5,6], and sit-ups [6] have been observed to begin to decline from the 20s or 30s in cohort studies. Age-related declines in physical strength, including sprint speed and jumping, have also been observed in sprint athletes [7]. Decline of physical strength leads to poor sports performance and increasing burdens in activities of daily living. Therefore, maintaining or enhancing physical strength in adults of all ages, not only elderly individuals but also young adults, is important to improve quality of life, and is expected to prevent sarcopenia [8], leading to an increase in healthy life expectancy.

Many previous studies have shown that exercise is effective in maintaining or improving physical strength [9–11]. Healthy nutrition also contributes to maintain and augment physical strength. Notably, protein supplementation combined with resistance training can improve muscle strength [12], and ingestion of several food ingredients, including astaxanthin [13], creatine [14], vitamin D [15] and probiotics [16] has also been shown to potentially maintain or improve physical strength. The milk fat globule membrane (MFGM), which is a structural membrane that covers the globules of triglycerides dispersed in an emulsion in milk, is one such food ingredient. In previous human studies compared with placebo (whole milk powder), MFGM ingestion combined with light aerobic exercise training improved agility in elderly individuals [17] and middle-aged adults 50 to 69 years old [18]. In addition, the combination of MFGM ingestion and light aerobic exercise improved muscle strength in men 31–48 years old [19]. Previous animal studies suggested that the effects of MFGM ingestion on physical performance derived from improved neuromuscular junction function [20–22]. The neuromuscular junction is the interface between motor neuron and the skeletal muscle, and the integrity and preservation of the neuromuscular junction is critical for maintaining muscle strength [23,24]. MFGM contains membrane-specific proteins, phospholipids, and sphingolipids [25], and Haramizu et al. suggesting that the beneficial effects of dietary MFGM combined with exercise are at least partly due to sphingomyelin, which is a type of sphingolipid found in animal cell membranes and one of the main components of MFGM [22]. In an in vitro assay, increased expression of Dok-7, which plays an essential role in neuromuscular junction formation [26],was found to be related to the presence of sphingomyelin [22].

Previous studies to investigate the effects of MFGM ingestion on agility or muscle strength [17,18] were designed using light aerobic exercise, but those outcomes are classified as instantaneous physical strength. We expected that the effects of MFGM ingestion on instantaneous physical strength would be enhanced when combined with instantaneous exercise training, but there is insufficient research data to support this hypothesis. To test our hypothesis, we designed a study in two phases. First, we chose power training as instantaneous exercise training and conducted a pilot study to calculate the appropriate number of study participants [27]. Then, in the present study, we evaluated the effects of 4 weeks of MFGM ingestion on muscle strength, agility and muscle power compared with placebo in healthy young adults. Because of the difficulty in quantifying MFGM content, we used sphingomyelin content (160 mg per day) as an indicator of MFGM content.

## 2. Materials and methods

### 2.1. Study design

The present study used a double-blind, placebo-controlled, parallel-group, randomized design. After acceptance into the study, participants were randomly allocated to either of two exercise groups, one combined with MFGM and the other with a placebo. All participants then started a 4-week exercise and supplement intervention. Body composition and physical performance were assessed before and after the intervention. The trial was conducted at Meiji Innovation Center, Meiji Co., Ltd. (Tokyo, Japan) between June and September 2023. The data were analyzed between October 2023 and May 2024. To set the appropriate number of participants, power analyses were conducted using R version 4.3.1 (R Project for Statistical Computing). Based on the results of the pilot study [27], we assumed that MFGM ingestion would induce differences of 0.10 Nm/kg lean body mass (LBM) in isokinetic knee extension and flexion strength changes with a standard deviation of 0.18, and a difference of 0.9 reps in the side-step repetition with a standard deviation of 1.5. The calculated sample sizes were 52 and 45 respectively, to attain 80% statistical power using a two-sided α of 0.05, so we decided to recruit approximate 100 participants (i.e. 50 participants per group). The present study was registered in the UMIN Clinical Trials Registry (UMIN000050921).

### 2.2. Ethics approval and consent to participate

The procedures in this study were approved by the Institutional Review Board of the Chiyoda Paramedical Care Clinic (Approval Number: MIJ23C2). It was conducted in accordance with ethical principles laid down by the Declaration of Helsinki and Ethical Guidelines for Medical and Health Research Involving Human Subjects (Ministry of Health, Labor and Welfare, Japan). Written informed consent was obtained from each participant before randomization.

### 2.3. Participants

Participants were recruited as study volunteers. All participants answered questionnaires about health history, physical activity (approximate frequency and time of standing, walking, running, cycling and playing other sports in a recent month), and habitual intake of dairy product and whey protein (approximate amount of intake in a recent week). Habitual intake of sphingomyelin from dairy products was calculated with reference to a prior literature [28]. Muscle strength declines clearly after around 50 years of age [1–3]; thus, the responsiveness of muscle strength gains following an intervention may differ between individuals younger or older than 50 years. In this study, we focused on adults under 50 years old. Accordingly, the inclusion criterion was 20–49 years. The exclusion criteria were: 1) current or previous history of significant diseases; 2) significant orthopedic injuries; 3) restriction of exercise, intense movement, or bathing due to medical reasons; 4) regular high intensity exercise; 5) milk or soy allergies; 6) habitual intake of more than 50 mg/day of sphingomyelin from dairy products; 7) habitual intake of whey protein; 7) smoker; 8) heavy drinker; 9) pregnant or breastfeeding; 10) BMI of 30 or more; 11) participant in other clinical studies; or 12) inappropriate for the study judged by the principal investigator for other reasons.

### 2.4. Randomization and blinding

Stratification based on sex was performed after eligibility assessment for study participation. Participants were divided into two groups: males or females. In each group, computer-generated random numbers using Microsoft Excel 2019 (Microsoft Corporation, WA, USA) were assigned to the participants who were then sorted and divided into two equal groups. The groups were randomly assigned to either MFGM or placebo by an individual who was not involved in the planning, enrollment, evaluation, intervention, or analysis. The participants, investigators, and all staff members involved in the trial were blinded to group allocation. The randomization code was opened after the study data were checked, collated, and finalized.

### 2.5. Supplementation protocol

Participants ingested either MFGM powder (SureStart^TM^ MFGM Lipid 70, Fonterra Co-operative Group Limited, Auckland, New Zealand) or a placebo powder dissolved in water daily for four weeks. The MFGM powder contained 0.2 g carbohydrate, 7.6 g protein, 1.7 g fat and 160 mg sphingomyelin, providing 46 kcal of energy per package. MFGM powder is made from whey protein concentrate. The sphingomyelin content was quantified using thin-layer chromatography conducted by Japan Food Research Laboratories (Tokyo, Japan). The placebo powder was made from whey protein isolate and maltodextrin, and contained 3.8 g carbohydrate, 7.6 g protein, 0.0 g fat and less than 5 mg (detection limit) of sphingomyelin, providing 46 kcal of energy per package. On the days of exercise training, the participants were instructed to consume a test powder within 60 minutes before each exercise training session. On the other days, they were instructed to consume a test powder anytime they wanted. The participants recorded the ingestion time of the test powder on a record sheet, and we checked the record sheet every day of exercise training. During the intervention period, participants were instructed to refrain from ingesting more dairy products than they did normally and to keep their diet the same as before the intervention.

### 2.6. Exercise training program

All participants were instructed to perform an exercise training program 3 days/week for 4 weeks at the MEGALOS sports club in Hachioji City (Tokyo, Japan). We selected power training in the exercise program to improve instantaneous physical strength. The exercise training program was composed of the following: leg press, chest press (3 sets of 20 reps at 30% of 1 repetition maximum, RM), countermovement jump (1 set of 10 reps), stepping (3 sets of 5 sec each), sit-ups (the repetition times of the baseline measurement in each participant). Participants were instructed to perform the movements as quickly as possible in each training program. The training program (form, load, speed, and number of repetitions) was supervised by the investigators at the sports club. The training loads of the leg press and chest press were set in reference to those of previous studies [9,29,30], and the number of repetitions was set as the number of times the move could be performed by a female, to maintain compliance in the present study.

### 2.7. Measurements

Physical strength tests and body composition assessments were performed at baseline and after 4 weeks of intervention by experienced staff members who were blinded to group allocation. An interval of one day or longer was implemented between the last training session and the post-intervention measurement. On the day before the measurement, participants were barred from drinking alcohol. On the day of the measurement, participants were barred from drinking alcohol and drinks containing caffeine (coffee, black tea, green tea or other nutritional drinks), but were not barred from consuming other foods or staying hydrated. Post-intervention, each participant was measured at the same time of day as the baseline measurement was made.

#### 2.7.1. Isokinetic knee extension/flexion strength and power

Peak torque and average power of isokinetic knee extensors and flexors were evaluated using an isokinetic dynamometer (Biodex System 4, Biodex Medical Systems, Inc. NY, USA) at an angular speed of 180°/s. Full knee flexion (start-position) was set to 100° and full knee extension was set to 10°. Following a familiarizing practice trial, participants extended and then flexed their right knee with full strength. The test was repeated 10 times with no interval and the best result was recorded.

#### 2.7.2. 1 repetition maximum (RM) test

Maximum strength was evaluated by 1RM strength tests on leg press using a leg press machine (MATRIX, Johnson Health Tech, Taichung City, Taiwan) and chest press using a Smith machine (TuffStuff Fitness International, Inc., CA, USA). Before the day of the baseline measurement, participants practiced the 1RM test. On the day of the baseline measurement, participants performed a warm-up of 3 repetitions with a load of approx. 50% of the preliminary 1RM, and one repetition with a load of approx. 90% of the preliminary 1RM. After warm-up, the load was increased in increments of 1–20 kg for the leg press or 1–6 kg for the chest press until only one successful repetition could be completed. One-minute rests were given between each test. The increase or decrease in the load continued until the participant was able to complete one repetition with the proper exercise technique. Post-intervention, participants performed the 1RM test in the same steps based on the baseline 1RM.

#### 2.7.3. Side-step test

Side-step test performance was evaluated as an indicator of agility. The side-step test is one of the physical fitness tests established by the Ministry of Education, Culture, Sports, Science and Technology-Japan [31]. Three lines were drawn at 100 cm intervals, and participants stood at a center line. Participants stepped 100 cm to the side and touched a line with the closest foot, stepped back to the center, and then stepped 100 cm to the other side and back to the center again for one complete cycle [32]. The number of times the lines were touched or crossed within 10 seconds was counted. The test was repeated 2 times at an interval of 2 minutes and the best result was recorded.

#### 2.7.4. Stepping test

Step test performance was evaluated as an indicator of agility. Participants stood on a mat with their legs in a parallel position. Participants stepped on the mat, without allowing their heels to touch the mat, as fast as possible for 5 seconds. The number of steps was counted using a step counting instrument (T.K.K.5301; Takei Scientific Instruments, Co. Ltd., Niigata, Japan) [33]. The test was repeated twice and the best result was recorded.

#### 2.7.5. Vertical jump test

Vertical jump height was evaluated as an indicator of muscle power. Participants were instructed to squat and then immediately jump as high as possible while keeping their arms crossed in front of the chest. Vertical jump height was measured using a 6-camera motion capture system (Opti Track, Acuity Inc., Tokyo, Japan). Participants were fit with two retro-reflective markers on their waist and the marker data were recorded at 240 Hz. The marker data were filtered using a fourth order low-pass Butterworth filter with a 20 Hz cutoff frequency. The test was repeated five times and the average value of three trials excluding the maximum and minimum values was recorded.

#### 2.7.6. Sit-ups test

The number of sit-ups that could be performed in 30 seconds was evaluated as an indicator of muscle power. The sit-ups test is one of the physical fitness tests established by the Ministry of Education, Culture, Sports, Science and Technology-Japan [31]. Participants laid supine on a mat with their hands lightly clasped and arms crossed in front of the chest. Both ankles were fixed, and knees were flexed at 90°. From the supine position, the upper body was raised until both elbows and thighs touched. Then, participants quickly returned to the supine position at the start. The movements were repeated as fast as possible for 30 seconds. The sit-ups test was performed once on each measurement day, considering the fatigue of the participants. In order to enhance accuracy of the sit-ups test, participants practiced the test before the baseline measurement day.

#### 2.7.7. Body composition assessments

A direct segmental multifrequency (5 kHz, 50 kHz and 250 kHz) bioelectrical impedance analysis (DSM-BIA) device using an 8-point tactile electrode system (InBody 430, InBody Co., Ltd., Seoul, Korea) [34] was used to measure body weight and lean body mass. Participants were measured wearing the same shirt and pair of shorts, which weighed 0.5 kg, so the weight adjustment for clothing was set to 0.5 kg.

### 2.8. Statistical analysis

All statistical analyses were performed by an independent organization (CPCC Co., Ltd., Tokyo, Japan) using Microsoft Excel 2019 (Microsoft Corporation, WA, USA) and IBM SPSS Statistics 26 (IBM Japan, Ltd., Tokyo, Japan). All values are expressed as mean ± standard deviation (SD) or standard error of the mean (SEM). An independent-samples *t*-test was used to compare differences between groups in participant characteristics at baseline, compliance rates for exercise training and consumption of test powders, and the interval between the last training session and the post-intervention measurement. To test differences between groups of post-intervention values, an analysis of covariance (ANCOVA) model with the baseline value as a covariate was carried out for physical strength and body composition data. A paired-samples *t*-tests was used to determine the intervention effect within each group. To assess the impact of sex on the effects of MFGM ingestion, a two-way analysis of variance (ANOVA) with group and sex as the between-participants factor was carried out for changes in values during the intervention period. For all statistical analyses, significance was set at *p* < 0.05. Additionally, a standardized mean difference for changes in values during the intervention period was determined by calculating Cohen’s *d* to assess the magnitude of the intervention’s effect.

## 3. Results

### 3.1. Participants

**Figure 1.**
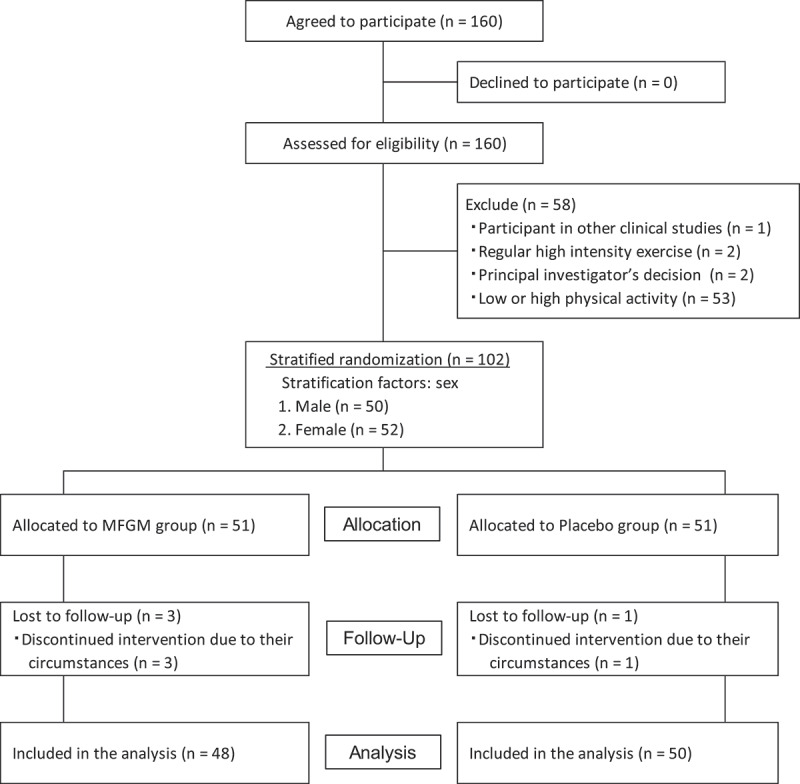
CONSORT flowchart for the human intervention study.

The participant flow through the protocol is shown in Figure 1. Five participants were excluded based on the exclusion criteria. From the 155 remaining participants, 50 males and 52 females were selected in ascending order of the difference from the median of physical activity based on metabolic equivalents (METs) [35], as determined by questionnaires. Then, 102 participants aged 20–49 were randomly allocated to the MFGM (*n* = 51) or Placebo (*n* = 51) group, with 25 males and 26 females in each group. Four participants discontinued the intervention due to their circumstances, and 98 participants completed the 4-week intervention. One participant in the Placebo group injured his ankle on the day of the post-intervention measurement, so he could not perform the 1 RM test, side step test, stepping test, and sit-ups test. The representative characteristics of the 98 participants measured at baseline are presented in Table 1; there were no significant differences between the two groups at baseline.

**Table 1.** Baseline participant characteristics.

	MFGM	Placebo	Group
Female/Male, n	23/25	26/24	–
Age, year	33.8 ± 8.3	33.9 ± 7.9	*t* (96) = −0.07, *p* = 0.947
Height, cm	166.1 ± 8.3	164.3 ± 8.7	*t* (96) = 1.03, *p* = 0.304
Body weight, kg	61.5 ± 10.9	60.7 ± 11.1	*t* (96) = 0.36, *p* = 0.721
BMI, kg/m^2^	22.2 ± 2.7	22.4 ± 2.9	*t* (96) = −0.34, *p* = 0.733
Lean body mass, kg	46.7 ± 9.1	45.3 ± 9.4	*t* (96) = 0.72, *p* = 0.473
Sphingomyelin intake from dairy products, mg/day	11.8 ± 11.2	10.6 ± 12.6	*t* (96) = 0.47, *p* = 0.640

Data are presented as mean ± SD; *n* = 48–50. MFGM, milk fat globule membrane. BMI, body mass index. An independent-samples *t*-test was used to compare differences in participant characteristics at baseline between groups.

### 3.2. Protocol compliance

The mean compliance (± SDs) for each group were as follows: exercise training compliance, 99.7 ± 2.4% (min. 83.3%) in the MFGM group and 99.5 ± 2.0% (min. 91.7%) in the Placebo group; supplementation intake compliance, 99.9 ± 0.5% (min. 96.4%) in the MFGM group and 99.4 ± 2.1% (min. 89.3%) in the Placebo group. There were no significant differences between groups in exercise training compliance (*t* (96) = 0.34, *p* = 0.737) or supplementation intake compliance (*t* (96) = 1.60, *p* = 0.113). We set the cutoff value of the compliance rate as 80% for analysis, but none of the participants who completed the intervention met the criteria.

The mean intervals (± SDs) between the last training session and the post-intervention measurement were 2.0 ± 0.9 days in the MFGM group and 2.0 ± 0.9 days in the Placebo group, there was no significant difference (*t* (1, 96) = 0.00, *p* = 1.000).

### 3.3. Physical strength tests

The peak torque and average power of isokinetic knee extensors and flexors at baseline and after the 4 wk intervention are presented as means ± SEMs in Table 2. According to the ANCOVA model, the peak torque and average power of isokinetic knee extensors after the 4 wk intervention in the MFGM group (2.23 ± 0.05 N･m/kg LBM and 3.78 ± 0.11 W/kg LBM, respectively) were significantly higher than those in the Placebo group (2.07 ± 0.06 N･m/kg LBM and 3.47 ± 0.12 W/kg LBM, respectively) when baseline values were used as covariates (*F* (1, 96) = 9.34, *p* = 0.003 and *F* (1, 96) = 5.68, *p* = 0.019, respectively). Other physical strength test values at baseline and after the 4 wk intervention are presented as means ± SEMs in Table 3. Leg press 1RM and sit-ups reps after the 4 wk intervention in the MFGM group (3.17 ± 0.07 kg/kg LBM and 20.1 ± 0.8 reps, respectively) were significantly higher than those in the Placebo group (3.02 ± 0.07 kg/kg LBM and 18.7 ± 0.6 reps, respectively) when baseline values were used as covariates (*F* (1, 95) = 8.59, *p* = 0.004 and *F* (1, 95) = 4.88, *p* = 0.030, respectively).

**Table 2.** Peak torque and average power of isokinetic knee extensors and flexors at baseline and after the 4 wk intervention.

	MFGM		Placebo		ANCOVA
	Baseline	4 wk	Baseline	4 wk	Group (4-wk values)
Peak torque, N･m/kg LBM					
Isokinetic knee extensors	2.16 ± 0.05	2.23 ± 0.05*	2.10 ± 0.06	2.07 ± 0.06	*F* (1,96) = 9.34, *p* = 0.003
Isokinetic knee flexors	1.03 ± 0.04	1.09 ± 0.04*	1.02 ± 0.04	1.06 ± 0.04*	*F* (1,96) = 0.30, *p* = 0.587
Average power, W/kg LBM					
Isokinetic knee extensors	3.53 ± 0.11	3.78 ± 0.11*	3.44 ± 0.11	3.47 ± 0.12	*F* (1,96) = 5.68, *p* = 0.019
Isokinetic knee flexors	1.35 ± 0.07	1.45 ± 0.06*	1.28 ± 0.06	1.38 ± 0.08*	*F* (1,96) = 0.01, *p* = 0.934

Data are presented as mean ± SEM; *n* = 48–50. MFGM, milk fat globule membrane. LBM, lean body mass. An analysis of covariance (ANCOVA) model with baseline values as a covariate was performed to test differences in post-intervention values for peak torque and average power of isokinetic knee extensors and flexors between groups. A paired-samples *t* test was used to determine the intervention effect within each group. *Significantly different from baseline within each group, *p* < 0.05.

**Table 3.** Physical strength test values at baseline and after the 4 wk intervention.

	MFGM		Placebo		ANCOVA
	Baseline	4 wk	Baseline	4 wk	Group (4-wk values)
1RM, kg/kg LBM					
Leg press	2.81 ± 0.07	3.17 ± 0.07*	2.77 ± 0.07	3.02 ± 0.07*	*F* (1,95) = 8.59, *p* = 0.004
Chest press	0.82 ± 0.03	0.85 ± 0.03*	0.82 ± 0.03	0.85 ± 0.03*	*F* (1,95) = 0.17, *p* = 0.681
Side-step test, reps	22.5 ± 0.7	23.9 ± 0.6*	21.9 ± 0.6	23.1 ± 0.5*	*F* (1,95) = 0.98, *p* = 0.324
Stepping test, reps	45.3 ± 1.1	50.1 ± 1.0*	45.6 ± 1.2	49.4 ± 1.2*	*F* (1,95) = 0.76, *p* = 0.386
Vertical jump height, m	0.32 ± 0.01	0.34 ± 0.01*	0.31 ± 0.01	0.33 ± 0.01*	*F* (1,94) = 0.00, *p* = 0.981
Sit-ups test, reps	16.5 ± 0.7	20.1 ± 0.8*	16.2 ± 0.6	18.7 ± 0.6*	*F* (1,95) = 4.88, *p* = 0.030

Data are presented as mean ± SEM; *n* = 47–49. MFGM, milk fat globule membrane. LBM, lean body mass. An analysis of covariance (ANCOVA) model with baseline values as a covariate was performed to test differences in post-intervention values for physical strength between groups. A paired-samples *t* test was used to determine the intervention effect within each group. *Significantly different from baseline within each group, *p* < 0.05.

There were no significant group × sex interactions and main effects of sex in the changes in physical strength test values during the intervention period (Table 4). Significant group effects were found for the changes during the 4 wk intervention in peak torque of isokinetic knee extensors (*F* (1, 96) = 9.16, *p* = 0.003), average power of isokinetic knee extensors (*F* (1, 96) = 5.21, *p* = 0.025), leg press 1RM (*F* (1, 95) = 7.61, *p* = 0.007), and sit-up reps (*F* (1, 95) = 4.61, *p* = 0.034). The effect size, measured by Cohen’s *d*, was *d* = 0.61 in peak torque of isokinetic knee extensors, *d* = 0.47 in average power of isokinetic knee extensors, *d* = 0.57 in leg press 1RM, and *d* = 0.44 in sit-up reps (Table 4).

**Table 4.** Changes in physical strength during the 4 wk intervention.

	MFGM	Placebo	Cohen’s d	*P* (ANOVA)		
				Group	Sex	Interaction
Peak torque, N･m/kg LBM						
Isokinetic knee extensors	0.071 ± 0.018	−0.024 ± 0.026	0.61	0.003	0.388	0.697
Isokinetic knee flexors	0.061 ± 0.014	0.047 ± 0.023	0.10	0.628	0.646	0.863
Average power, W/kg LBM						
Isokinetic knee extensors	0.245 ± 0.063	0.031 ± 0.067	0.47	0.025	0.854	0.465
Isokinetic knee flexors	0.103 ± 0.041	0.108 ± 0.041	0.02	0.938	0.765	0.138
1RM, kg/kg LBM						
Leg press	0.363 ± 0.031	0.250 ± 0.026	0.57	0.007	0.864	0.843
Chest press	0.029 ± 0.006	0.026 ± 0.005	0.09	0.688	0.861	0.157
Side-step test, reps	1.4 ± 0.3	1.2 ± 0.3	0.12	0.556	0.561	0.156
Stepping test, reps	4.8 ± 0.7	3.9 ± 0.7	0.18	0.403	0.668	0.955
Vertical jump height, m	0.018 ± 0.003	0.019 ± 0.003	0.04	0.888	0.632	0.476
Sit-ups test, reps	3.5 ± 0.4	2.5 ± 0.3	0.44	0.034	0.885	0.679

Data are presented as mean ± SEM; *n* = 47–50. MFGM, milk fat globule membrane. LBM, lean body mass. A two-way analysis of variance (ANOVA) with group and sex as the between-participants factor was performed for changes in values during the intervention period. The standardized mean difference for changes in values during the intervention period was determined by calculating Cohen’s *d* to assess the magnitude of the intervention’s effect.

### 3.4. Body composition assessments

Body weight and lean body mass at baseline and after the 4 wk intervention are presented in Table 5. There were no significant group differences in body weight and lean body mass after the 4 wk intervention in the ANCOVA model (*F* (1, 96) = 0.58, *p* = 0.448 and *F* (1, 96) = 0.41, *p* = 0.524, respectively). No significant main effects of group or sex, or of group × sex interactions were found for the changes during the 4 wk intervention in body weight and lean body mass (Table 6).

**Table 5.** Body weight and lean body mass at baseline and after the 4 wk intervention.

	MFGM		Placebo		ANCOVA
	Baseline	4 wk	Baseline	4 wk	Group (4-wk values)
Body weight, kg	61.5 ± 1.6	61.7 ± 1.6*	60.7 ± 1.6	60.8 ± 1.6	*F* (1,96) = 0.58, *p* = 0.448
Lean body mass, kg	46.7 ± 1.3	46.7 ± 1.3	45.3 ± 1.3	45.5 ± 1.3	*F* (1,96) = 0.41, *p* = 0.524

Data are presented as mean ± SEM; *n* = 48–50. MFGM, milk fat globule membrane. An analysis of covariance (ANCOVA) model with baseline values as a covariate was performed to test differences in post-intervention values for body weight and lean body mass data between groups. A paired-samples *t* test was used to determine the intervention effect within each group. *Significantly different from baseline within each group, *p* < 0.05.

**Table 6.** Changes in body composition during the 4 wk intervention.

	MFGM	Placebo	Cohen’s d	*P* (ANOVA)		
				Group	Sex	Interaction
Body weight, kg	0.24 ± 0.10	0.09 ± 0.15	0.16	0.450	0.608	0.409
Lean body mass, kg	0.04 ± 0.14	0.20 ± 0.14	0.16	0.462	0.430	0.598

Data are presented as mean ± SEM; *n* = 48–50. MFGM, milk fat globule membrane. A two-way analysis of variance (ANOVA) with group and sex as the between-participants factor was performed for changes in values during the intervention period. The standardized mean difference for changes in values during the intervention period was determined by calculating Cohen’s *d* to assess the magnitude of the intervention’s effect.

## 4. Discussion

We investigated the effects of daily MFGM ingestion during 4 weeks of power training on instantaneous physical strength – muscle strength, agility, and muscle power – in young adults compared with placebo ingestion. The major findings in the present study are that MFGM ingestion combined with power training improves muscle strength and power in young adults.

In the present study, the peak torque of isokinetic knee extensors and leg press 1RM after the 4 wk intervention in the MFGM group were significantly higher than those in the Placebo group. Moreover, the changes of peak torque of isokinetic knee extensors and leg press 1RM in the MFGM group were greater than those in the Placebo group. The results indicated that MFGM ingestion significantly improved knee and leg extension strength. The differences of increase rates during the intervention in knee and leg extension strength between groups were 4.7% and 3.8%, respectively. The previous meta-analysis in adults revealed that high-dose protein supplementation (≥0.5 g/kg body weight/day) combined with resistance training induced 4.3% increase in muscle strength compared with control group [12], which is similar to the impacts of MFGM ingestion in the present study. In a previous study, Soga et al. demonstrated that MFGM ingestion combined with aerobic exercise improved isokinetic leg extension strength compared with placebo (whole milk powder) in healthy males aged 31–48 [19], consistent with the results of the present study. MFGM ingestion combined with aerobic exercise induced approximately 5% improvement in leg extension strength compared to the placebo group in Soga’s study [19], and MFGM ingestion combined with power training induced approximately 4% improvement in knee and leg extension strength compared to placebo in the present study. The two studies showed that the same level of improvement in muscle strength induced by MFGM ingestion, suggesting that the effects of MFGM ingestion on muscle strength may not be affected by different types of exercise. However, it should be noted that previous meta-analyses showed that high load resistance training is more effective for increasing muscle strength than low load resistance training [10,36,37]. While the present training program required quick movements, the weight load was low (body weight training or 30% of 1RM). The effects of MFGM ingestion on muscle strength when combined with high load resistance training need to be verified in the future. Unlike the results for knee extension strength, knee flexion strength in the MFGM group did not improve compared with the Placebo group. The training protocol in the present study added resistance to knee extension movements (leg press and counter-movement jump), but not to knee flexion movements. This suggests that the effect of MFGM ingestion on muscle strength may be affected by how the muscle is used during exercise, muscle activity over a particular level during exercise training may be required to obtain the benefits of MFGM supplementation. To verify the hypothesis, studies are needed to examine the effects of MFGM on muscle strength without exercise intervention.

In the present study, exercise training significantly improved agility in healthy young adults, but MFGM ingestion did not. In previous studies on the elderly, MFGM ingestion combined with light aerobic exercise training improved agility. The discrepancy may be due to differences in the age of the participants. The two reviews discuss the tendencies of fast-to-slow muscle fiber-type transitions in aged skeletal muscle [38,39]. Moreover, a higher susceptibility of fast-twitch fibers to muscular atrophy in aged human skeletal muscles were observed in the studies that analyzed muscle samples from both young and old individuals [7,40]. It is possible that the effects of MFGM ingestion on agility are more sensitive in older adults, who have age-related atrophy of fast-twitch muscle fibers. On the other hand, MFGM ingestion improved muscle power during knee extension and the sit-ups test as an indicator of muscle power. Power is defined as producing the greatest amount of force in the shortest possible time. A previous study in female handball players demonstrated that muscle power for the shoulder is positively correlated with ball-throwing speed [41]. Muscle power is a product of force and velocity, i.e. strength and speed, so increased muscle strength and/or agility could contribute to increase muscle power. The results of the present study suggested that muscle strength has a greater effect on improving muscle power in the MFGM group.

Improved neurological adaptation in skeletal muscles has been recognized to make a larger contribution to early strength gains during isotonic strength training compared to muscle hypertrophy [42], particularly during the first 4 weeks [43]. The present study showed that 4-wk daily MFGM ingestion combined with power training improved muscle strength and power in healthy young adults without muscle mass increases, suggesting that MFGM ingestion induced improvement of neurological adaptation. This hypothesis is supported by the results of a previous study in which MFGM ingestion increased the root mean square (RMS) of surface electromyography (EMG) during leg extension exercise in males aged 31–48 [19]. Surface EMG comprises the sum of the electrical contributions made by the active motor units, and its amplitude is related to the net motor unit activity, such as the recruitment of the active motor units [44]. Increments of motor unit recruitment during neurological adaptation lead to increased RMS of the surface EMG. A previous animal study also suggested that MFGM ingestion combined with exercise has beneficial effects on muscle strength by stimulating the pathway involving “nervous system development” in the skeletal muscle [22]. It is possible that sphingomyelin, which is one of the main components of MFGM, contribute to a mechanism through which MFGM ingestion combined with exercise improves neurological adaptation. Dietary sphingomyelin is hydrolyzed into ceramide and then sphingosine, which is subsequently absorbed in the mucosal cells [45]. Absorbed sphingosine is metabolized into sphingosine-1-phosphate (S1P) or resynthesized into ceramide and then sphingomyelin [45]. Haramizu et al. showed that *Dok-7* mRNA expression is upregulated in differentiating C2C12 cells via milk-derived sphingomyelin [22]. Overexpression of Dok-7 in skeletal muscle enhances neuromuscular transmission with significant enlargement and ultrastructural alterations of neuromuscular junction in mice [46]. Furthermore, previous studies have suggested that S1P enhances spontaneous transmitter release at the neuromuscular junction in frogs [47] and *Caenorhabditis elegans* [48].

The present study includes the following limitations: 1) We did not evaluate food intake and physical activity during the intervention period; therefore, the influence of daily diet, e.g. protein intake, and physical activity, e.g. playing sports, during the intervention period could not be excluded. 2) Neurological factors improve dramatically and contribute most significantly to strength gains during the first 4 weeks of a resistance training program; after 4 weeks, neurological factors continue to improve slowly [43]. Therefore, the effects of MFGM ingestion combined with long-term exercise training may differ from the results of the present study, including the impact of morphological adaptations following improved neurological adaptations. 3) Both MFGM and placebo powder contained 7.6 g whey protein, so the results include the effect of protein plus MFGM compared with protein alone. Finally, the study design did not include a nonintervention group or an MFGM supplementation alone group, and that may limit the interpretation of the study results. It is not clear if the increases in muscle strength and power were due to MFGM supplementation alone or to the interactive effect of MFGM supplementation and exercise.

## 5. Conclusion

In conclusion, the present study has demonstrated that four weeks of MFGM ingestion combined with power training increased knee and leg extension strength, knee extension power, and sit-ups test values, but not agility in young adults compared with placebo. Therefore, MFGM supplementation plus exercise is effective to improve instantaneous physical strength, such as muscle strength and power. Extension of healthy life expectancy and improvement in quality of life is an important global issue. Physical strength is a key factor contributing to this and we are encouraged that the MFGM supplementation strategy proposed in the present study will improve physical strength, which gradually declines with age after adulthood.

## Data Availability

The data that supports the findings of this study are available in the supplementary file of this article.
